# Thermo-hydraulic and second-law analysis of a shell-and-tube heat exchanger using water, propylene glycol, and glycerol mixtures in the laminar–transition regime

**DOI:** 10.1038/s41598-026-61908-6

**Published:** 2026-07-20

**Authors:** Amr M. Hassaan

**Affiliations:** Mechanical Eng. Department, Higher Technological Institute (HTI), 10th, Ramadan City, Egypt

**Keywords:** Shell-and-tube heat exchanger, Thermo-hydraulic performance, Nusselt number, Reynolds number, Entropy generation, Exergy efficiency, Propylene glycol, Glycerol, Empirical correlation, Energy science and technology, Engineering, Mathematics and computing, Physics

## Abstract

This study presents a comprehensive experimental investigation of the thermo-hydraulic and second-law performance of a shell-and-tube heat exchanger operating under counter-flow conditions. Water, propylene glycol/water (PG/water), and glycerol/water mixtures at different concentrations were employed as working fluids to evaluate the influence of thermophysical properties on heat-transfer, hydraulic, and exergy characteristics. A total of 105 experimental runs were conducted over a Reynolds number range of 400–4800, covering laminar and transitional flow regimes. The overall heat transfer coefficient, pressure drop, Nusselt number, friction factor, entropy generation, and exergy efficiency were systematically analyzed and compared under identical operating conditions. The results demonstrated that increasing Reynolds number significantly enhances heat-transfer performance, resulting in higher overall heat-transfer coefficients and Nusselt numbers. However, this improvement is accompanied by increased pressure losses and entropy generation, reflecting the thermodynamic trade-off between heat-transfer enhancement and irreversibility. Among the investigated fluids, water exhibited the highest thermal and exergy performance, whereas glycerol/water mixtures produced the lowest performance due to their higher viscosity and associated flow resistance. To provide practical predictive tools, empirical correlations were developed for both the Nusselt number and friction factor based on the complete experimental dataset. The proposed correlations were expressed as Nu = 2.43*Re^0.30*Pr^0.094 and f = 5.47 Re^-0.62*Pr^-0.08, showing satisfactory agreement with the experimental measurements. Validation against established literature correlations confirmed the capability of the proposed models to predict thermal and hydraulic performance within the investigated Reynolds number range. The novelty of the present work lies in the comparative experimental assessment of water, propylene glycol/water, and glycerol/water mixtures under identical operating conditions, together with the simultaneous evaluation of thermal, hydraulic, and second-law performance metrics and the development of predictive correlations based on an extensive experimental database. The findings provide reliable benchmark data and engineering correlations that can support the design, optimization, and exergy-based evaluation of heat exchangers operating with viscous heat-transfer fluids in industrial thermal systems.

## Introduction

Heat exchangers have been extensively studied due to their critical role in thermal systems, particularly in industrial applications where efficient energy transfer is essential^1^. Shell-and-tube heat exchangers remain among the most commonly used configurations because of their mechanical reliability and ability to operate under a wide range of conditions^2–4^. Early foundational studies established that heat transfer performance is primarily governed by dimensionless parameters such as Reynolds and Prandtl numbers, which encapsulate the effects of flow regime and thermophysical properties^5,6^. Several experimental studies have been conducted in recent years to improve the thermal and hydraulic performance of shell-and-tube heat exchangers. Gugulothu and Sanke^7^ experimentally investigated the heat transfer characteristics of shell-and-tube heat exchangers equipped with different baffle configurations and reported that helical baffles significantly enhanced the overall heat transfer coefficient while reducing pressure drop compared with conventional segmental baffles. Similarly, Sameer et al.^8^ examined the thermal performance of a segmental-baffled shell-and-tube heat exchanger using Al₂O₃ nanofluids and observed substantial improvements in heat exchanger effectiveness with increasing nanoparticle concentration, although accompanied by higher flow resistance. Yılmaz et al.^9^ experimentally studied the use of Al₂O₃-water nanofluids in a mini-channel shell-and-tube heat exchanger and found that heat transfer enhancement became more pronounced in the transition and turbulent flow regimes, while friction losses increased with nanoparticle loading. Bhattad et al.^10^ experimentally investigated the influence of nanoparticle shape and size on the thermal and hydraulic performance of plate evaporators using hybrid nanofluids, reporting noticeable improvements in heat transfer characteristics. More recently, Khan et al.^11^ performed a combined experimental and numerical investigation of shell-and-tube heat exchangers employing round and hexagonal tubes and demonstrated that tube geometry plays a significant role in augmenting heat transfer performance. Rostami et al.^12^ investigated the influence of novel inner-tube cross-sectional geometries on the performance of double-pipe heat exchangers and demonstrated that geometric modifications can significantly improve heat-transfer rates while affecting pressure-drop characteristics. Similarly, Kong et al.^13^ numerically examined heat transfer and flow behavior in S-shaped miniature cooling channels for gas turbine blade applications, highlighting the strong influence of Reynolds number and channel configuration on thermal performance and flow resistance. Wang et al.^14^ studied the effect of internal roughness on cooling performance in laid-back fan-shaped cooling holes and reported that surface roughness can substantially alter flow structures and reduce heat-transfer effectiveness due to increased flow non-uniformity and mixing losses. In another experimental study, Bhattad et al.^15^ examined the hydrothermal performance of different alumina-based hybrid nanofluids in a plate heat exchanger and showed that the particle composition strongly affects both heat transfer enhancement and pressure-drop characteristics. Furthermore, Chen et al.^16^ performed a thermo-hydraulic analysis of double-layer microchannel heat sinks and demonstrated that geometric parameters strongly affect heat-transfer enhancement and overall thermal-fluid performance. Collectively, these studies emphasize the importance of flow conditions, geometry, and thermo-hydraulic interactions in determining heat-transfer performance.

Recent investigations have increasingly focused on advanced heat transfer enhancement techniques through the utilization of nanofluids, hybrid nanofluids, and innovative surface geometries. Bhattad et al.^17^ investigated the effect of particle ratio on the hydrothermal performance of plate heat exchangers and demonstrated that an optimum nanoparticle ratio can simultaneously improve thermal performance while limiting hydraulic penalties. Ahmad et al.^18^ experimentally and numerically evaluated the thermo-hydrodynamic performance of a double-dimpled corrugated tube employing both single and hybrid nanofluids and reported significant enhancement in heat transfer characteristics accompanied by acceptable hydraulic penalties. In another study, the thermal-hydraulic performance and flow behavior within a curved trapezoidal corrugated channel equipped with E-shaped baffles were examined using hybrid nanofluids, demonstrating remarkable improvements in thermal performance owing to intensified secondary flow structures and enhanced mixing mechanisms^19^. Furthermore, advanced numerical analyses were conducted to assess nanofluid flow through corrugated minichannels, where detailed thermo-hydrodynamic evaluations revealed substantial augmentation in heat transfer rates resulting from flow disruption and boundary layer thinning effects^20^.

Additional studies have investigated the influence of complex geometrical modifications on the thermal performance of heat transfer systems. The advanced thermo-hydrodynamic characteristics of nanofluids flowing through helically featured straight pipes were numerically assessed, indicating that helical surface features can effectively promote fluid mixing and improve thermal efficiency^21^. Similarly, comprehensive thermo-hydraulic assessments of helical pipes with various jacket configurations using both single-phase and hybrid nanofluids demonstrated considerable enhancement in overall heat transfer performance while maintaining reasonable pressure-drop levels^22^. Moreover, computational investigations on Al₂O₃/water nanofluids flowing through serpentine tube heat exchangers with varying straight-section lengths highlighted the strong influence of geometric parameters on heat transfer augmentation and flow behavior^23^. Collectively, these studies confirm the growing interest in combining advanced geometrical modifications with enhanced working fluids to achieve superior thermo-hydrodynamic performance. Recent studies have highlighted the growing importance of thermodynamic, thermal-hydraulic, and exergy-based analyses for improving the performance of heat transfer systems. Sundar et al.^24^ experimentally investigated the second-law efficiency and thermal entropy generation of SiO₂ nanofluids based on a 30:70 glycerol–water mixture in a thermosyphon flat-plate collector. Their results demonstrated that the incorporation of nanoparticles improved thermal performance and exergy efficiency while reducing entropy generation under optimal operating conditions. In addition, Sarkar and Bhattad^25^ presented a comprehensive review of nanofluid-based heat transfer enhancement techniques, highlighting the importance of thermo-hydraulic optimization and the need for reliable performance evaluation under practical operating conditions. In a subsequent study, Prajapati et al.^26^ applied advanced exergy analysis to a shell-and-tube heat exchanger and revealed that conventional exergy analysis may underestimate the sources of thermodynamic inefficiencies. Their findings showed that advanced exergy methods provide a more comprehensive understanding of avoidable and unavoidable exergy destruction, thereby offering valuable guidance for heat exchanger optimization.

Furthermore, a comprehensive review conducted by Marzouk et al.^27^ summarized the major heat transfer enhancement techniques employed in shell-and-tube heat exchangers, including passive, active, and compound methods. The review emphasized that modifications in flow geometry, surface characteristics, and working fluids can substantially improve thermal performance, although the associated hydraulic penalties must be carefully considered. With regard to alternative heat transfer fluids, Satti et al.^28^ investigated the thermal conductivity of propylene glycol-based nanofluids and compared the experimental results with available predictive correlations. Their study confirmed that thermal conductivity increases with nanoparticle concentration and temperature, indicating the potential applicability of propylene glycol-based fluids in advanced thermal systems. Moreover, Gu et al.^29^ examined the heat transfer characteristics of propylene glycol–water mixtures on the shell side of a spiral-wound heat exchanger. The authors reported that increasing glycol concentration significantly affects heat transfer behavior due to changes in viscosity and thermophysical properties, highlighting the importance of selecting appropriate mixture concentrations for efficient heat exchanger operation. Collectively, these studies demonstrate the increasing interest in evaluating alternative working fluids and advanced thermodynamic performance indicators.

Although several studies have investigated the thermo-hydraulic and exergy performance of glycol-based fluids, relatively few studies have reported direct experimental comparisons of water, propylene glycol/water, and glycerol/water mixtures in shell-and-tube heat exchangers under identical operating conditions over the Reynolds number range of 400–4800. Furthermore, limited attention has been given to the simultaneous evaluation of thermal, hydraulic, and second-law performance indicators within a single experimental framework. Therefore, the present work provides a comprehensive experimental assessment of these fluids based on 105 experimental runs. In addition, empirical correlations are developed to predict the thermo-hydraulic behavior of the investigated mixtures while accounting for the influence of fluid properties and operating conditions.

## Experimental setup

**Fig. 1:**
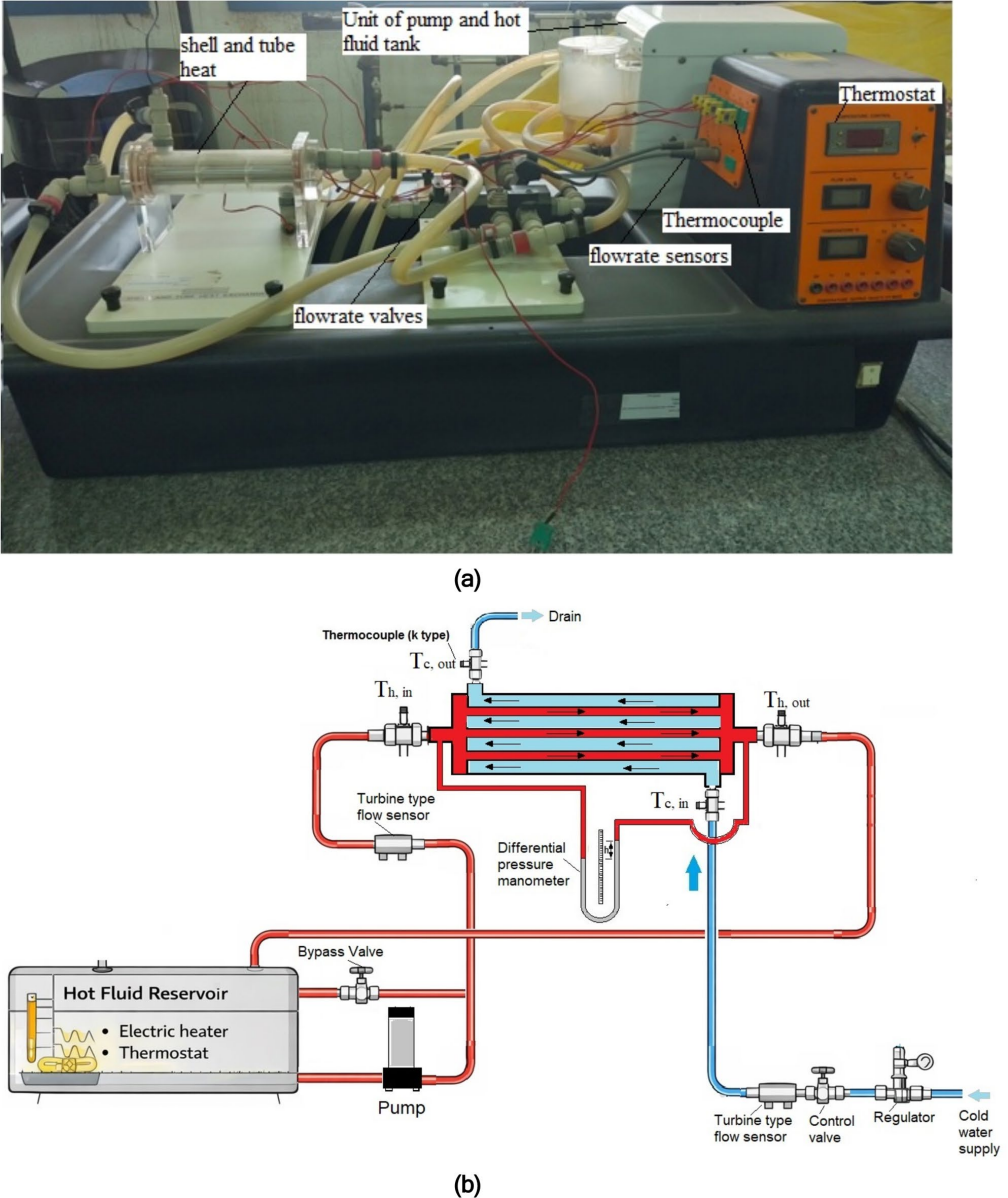
**a**- photo of the actual experimental setup **b**- schematic diagram of the test rig.

The experimental investigation was carried out using a shell-and-tube heat exchanger module (HT33) connected to a service unit (HT30X) both produced by Armfield company. Figure 1a shows a photographic presentation of the experimental test rig. The heat exchanger consists of seven parallel stainless-steel tubes enclosed within a transparent acrylic shell, allowing visual observation of the flow configuration. The hot fluid flows through the tube side, while cold water is circulated through the shell side in a counter-flow arrangement as shown in the schematic diagram in Fig. 1b. This configuration ensures a higher thermal driving force along the heat exchanger length, improving heat transfer performance compared to parallel flow.

The geometrical characteristics of the test section are as follows: the tubes have an outer diameter of 6.35 mm, an inner diameter of 5.15 mm, and an effective heat transfer length of 144 mm. There are two transverse baffles in the shell that raise the fluid’s velocity and, consequently, the rate of heat transfer. The outer surface area of the tubes is used as the reference area for calculating the overall heat transfer coefficient. The hot fluid is heated in a well-insulated reservoir equipped with an electric heater and a thermostat, which maintains the desired inlet temperature. The heated fluid is circulated through the tube side using a gear pump. A bypass valve is installed to regulate the flow rate and to stabilize the system before entering the test section. The cold fluid, which is tap water, flows through the shell side. Its flow rate is controlled using a control valve and monitored by a turbine-type flow sensor. The cold-side flow rate is maintained constant at 2.0 L/min for all experimental runs. The flow rate of hot and cold fluids can be changed via valves on the unit within a range of 0.5 to 3.5 L/minute.

Temperature measurements are obtained using calibrated thermocouples (type k) installed at the inlet and outlet of both hot and cold streams, denoted as $\:{T}_{h,in}$, $\:{T}_{h,out}$, $\:{T}_{c,in}$, and $\:{T}_{c,out}$, respectively. The volumetric flow rates of both hot and cold fluids are measured using turbine-type flow meters. The pressure drop across the tube side is measured using a U-tube differential manometer filled with mercury, connected between the inlet and outlet of the hot fluid line.

All experiments are conducted under steady-state conditions. The system is allowed to stabilize before recording measurements, ensuring that temperature and flow readings remain constant over time.

### Preparation of working fluids

Seven different working fluids were used in the present study, including pure water, glycerol/water mixtures, and propylene glycol (PG)/water mixtures. The mixtures were prepared based on mass fraction (wt%). Distilled water was used as the base fluid for all mixtures to ensure consistency and to avoid the influence of impurities on thermophysical properties. Glycerol/water mixtures with concentrations of 10 wt%, 20 wt%, and 30 wt% were prepared. The required mass of glycerol and water was calculated using:

1 $$\:\mathrm{wt}\mathrm{\%}=\frac{{m}_{solute}}{{m}_{solution}}\times\:100$$

For each mixture, the appropriate mass of glycerol was measured using a digital balance with high accuracy. Distilled water was then added gradually to achieve the desired concentration. The mixture was stirred continuously using a magnetic stirrer for sufficient time to ensure complete homogeneity. Care was taken to avoid air bubble formation during mixing.

Similarly, propylene glycol/water mixtures were prepared at concentrations of 10 wt%, 20 wt%, and 30 wt%.

The same procedure was followed:

- Accurate weighing of propylene glycol.
- Gradual addition of distilled water.
- Continuous mixing until a uniform solution is obtained.

All prepared mixtures were stored in sealed containers to prevent contamination and evaporation. Before each experimental run, the fluid was gently mixed to ensure uniform properties. The thermophysical properties of water, glycerol/water mixtures, and propylene glycol/water mixtures were evaluated at the bulk mean fluid temperature using published thermophysical-property data and correlations available in the literature^30,31^. The corresponding values employed in the calculations are summarized in Table 1.

**Table 1:** Fluid properties table.

Fluid	wt%	T (°C)	ρ (kg/m³)	cp. (J/kg.K)	µ(Pa.s)	k (W/m.K)
**Water**	0	60	983.2	4182	0.000467	0.653
	0	70	977.8	4187	0.000404	0.664
	0	80	971.8	4194	0.000355	0.672
**Glycerol/Water**	10	60	1017	4010	0.00074	0.427
	10	70	1011	4030	0.0006	0.431
	10	80	1005	4050	0.0005	0.435
	20	60	1039	3870	0.00105	0.404
	20	70	1032	3895	0.00083	0.409
	20	80	1025	3920	0.00068	0.414
	30	60	1060	3730	0.00155	0.381
	30	70	1052	3760	0.00118	0.387
	30	80	1044	3790	0.00092	0.393
**Propylene Glycol/Water**	10	60	1000	4120	0.00067	0.486
	10	70	988	4140	0.00054	0.491
	10	80	976	4160	0.00045	0.496
	20	60	1014	4040	0.00094	0.462
	20	70	1009	4060	0.00074	0.468
	20	80	1005	4080	0.0006	0.474
	30	60	1027	3960	0.00134	0.438
	30	70	1022	3985	0.00102	0.445
	30	80	1017	4010	0.0008	0.452

### Experimental operating conditions

The experiments were conducted under the following conditions:

- Hot fluid inlet temperatures: 60 °C, 70 °C, and 80 °C.
- Hot fluid flow rates: 1.0, 1.5, 2.0, 2.5, and 3.0 L/min.
- Cold water flow rate: constant at 2.0 L/min.
- Ambient temperature: approximately 25 °C.

A total of 105 experimental runs were performed covering all combinations of fluids, temperatures, and flow rates.

## Data reduction and governing equations

The experimental data were reduced using the following governing equations to evaluate the thermal, hydraulic, thermo-hydraulic, and second-law performance of the counter-flow shell-and-tube heat exchanger.

### Thermal performance analysis

The heat transfer rate released by the hot fluid was determined from^1^:

2 $$\:\begin{array}{cccc}&\:{Q}_{h}={\dot{m}}_{h}{c}_{p,h}({T}_{h,in}-{T}_{h,out})&\:&\:\end{array}$$

Similarly, the heat transfer rate gained by the cold fluid was calculated as^1^:

3 $$\:\begin{array}{cccc}&\:{Q}_{c}={\dot{m}}_{c}{c}_{p,c}({T}_{c,out}-{T}_{c,in})&\:&\:\end{array}$$

To reduce the effect of experimental heat losses and measurement uncertainty, the effective heat transfer rate used in the present analysis was taken as the arithmetic average of the hot- and cold-side values:

4 $$\:\begin{array}{cccc}&\:Q=\frac{{Q}_{h}+{Q}_{c}}{2}&\:&\:\end{array}$$

The logarithmic mean temperature difference (LMTD) was then calculated using^1^:

5 $$\:\begin{array}{cccc}&\:{\Delta\:}{T}_{lm}=\frac{{\Delta\:}{T}_{1}-{\Delta\:}{T}_{2}}{\mathrm{ln}\left(\frac{{\Delta\:}{T}_{1}}{{\Delta\:}{T}_{2}}\right)}&\:&\:\end{array}$$

$$\:{\Delta\:}{T}_{1}={T}_{h,in}-{T}_{c,out,\:\:\:}{\Delta\:}{T}_{2}={T}_{h,out}-{T}_{c,in}$$

The overall heat transfer coefficient based on the outer tube surface area was then obtained from^32^:

6 $$\:\begin{array}{cccc}&\:{U}_{o}=\frac{Q}{{A}_{o}{\Delta\:}{T}_{lm}}&\:&\:\end{array}$$

$$\:{A}_{o}=n\pi\:{d}_{o}L,\:n\dots\:no.\:of\:tubes$$

### Hydraulic performance analysis

The pressure drop of the hot working fluid flowing through the tube side was measured using a mercury differential U-tube manometer connected between the inlet and outlet of the heat exchanger. Accordingly, the tube-side pressure drop was evaluated as^33^:

7 $$\:\begin{array}{cccc}&\:{\Delta\:}P=({\rho\:}_{Hg}-{\rho\:}_{h})g{\Delta\:}h&\:&\:\end{array}$$

where $\:{\rho\:}_{Hg}$ is the density of mercury, $\:{\rho\:}_{h}$ is the density of the hot working fluid, $\:g$ is the gravitational acceleration, and $\:{\Delta\:}h$ is the measured manometer height difference.

The Reynolds number of the hot working fluid was determined using^34^:

8 $$\:\begin{array}{cccc}&\:R{e}_{h}=\frac{{\rho\:}_{h}{V}_{h}{d}_{i}}{{\mu\:}_{h}}&\:&\:\end{array}$$

$\:{V}_{h}\dots\:..$The mean velocity of the hot fluid inside the tubes.

For comparative evaluation of thermal performance, the Nusselt number was defined based on the experimentally determined overall heat transfer coefficient:

9 $$\:\begin{array}{cccc}&\:Nu=\frac{{U}_{o}D}{{K}_{h}}&\:&\:\end{array}$$

where Uo is the experimentally determined overall heat transfer coefficient, D is the characteristic tube diameter, and k is the thermal conductivity of the working fluid. Since all experiments were conducted using the same heat exchanger geometry, identical shell-side fluid, and similar shell-side operating conditions, the calculated Nusselt number was employed as a dimensionless indicator of the overall thermal performance of the investigated fluids.

while the Prandtl number was calculated from^33^:

10 $$\:\begin{array}{cccc}&\:P{r}_{h}=\frac{{c}_{p,h}{\mu\:}_{h}}{{k}_{h}}&\:&\:\end{array}$$

The experimental Darcy friction factor was evaluated based on the measured pressure drop as^33^:

11 $$\:\begin{array}{cccc}&\:{f}_{h}=\frac{2{\Delta\:}P{\hspace{0.17em}}{d}_{i}}{{\rho\:}_{h}L{V}_{h}^{2}}&\:&\:\end{array}$$

where $\:L$ is the effective tube length.

### Second-law analysis

The entropy rate change of the hot stream was calculated from^34^:

12 $$\:\begin{array}{cccc}&\:{\dot{S}}_{h}={\dot{m}}_{h}{c}_{p,h}\mathrm{l}\mathrm{n}\left(\frac{{T}_{h,out,K}}{{T}_{h,in,K}}\right)&\:&\:\end{array}$$

Similarly, the entropy rate change of the cold stream was determined as^34^:

13 $$\:\begin{array}{cccc}&\:{\dot{S}}_{c}={\dot{m}}_{c}{c}_{p,c}\mathrm{l}\mathrm{n}\left(\frac{{T}_{c,out,K}}{{T}_{c,in,K}}\right)&\:&\:\end{array}$$

Accordingly, the total entropy generation rate of the heat exchanger was evaluated by^34^:

14 $$\:\begin{array}{cccc}&\:{\dot{S}}_{gen}={\dot{S}}_{h}+{\dot{S}}_{c}&\:&\:\end{array}$$

where all temperatures in Eqs. (14) -(19) were expressed in Kelvin.

The exergy destruction rate was then obtained using the Gouy–Stodola theorem^34^:

15 $$\:\begin{array}{cccc}&\:{\dot{E}}_{d}={T}_{0}{\dot{S}}_{gen}&\:&\:\end{array}$$

where $\:{T}_{0}$ is the ambient dead-state temperature.

The exergy decrease of the hot stream was evaluated from^34^:

16 $$\:\begin{array}{cccc}&\:{\dot{E}}_{x,h}={\dot{m}}_{h}{c}_{p,h}\left[({T}_{h,in,K}-{T}_{h,out,K})-{T}_{0}\mathrm{l}\mathrm{n}\left(\frac{{T}_{h,in,K}}{{T}_{h,out,K}}\right)\right]&\:&\:\end{array}$$

Likewise, the exergy gain of the cold stream was calculated as^31^:

17 $$\:\begin{array}{cccc}&\:{\dot{E}}_{x,c}={\dot{m}}_{c}{c}_{p,c}\left[({T}_{c,out,K}-{T}_{c,in,K})-{T}_{0}\mathrm{l}\mathrm{n}\left(\frac{{T}_{c,out,K}}{{T}_{c,in,K}}\right)\right]&\:&\:\end{array}$$

The exergy efficiency of the heat exchanger was then defined as^31^:

18 $$\:\begin{array}{cccc}&\:{\eta\:}_{ex}=\frac{{\dot{E}}_{x,c}}{{\dot{E}}_{x,h}}&\:&\:\end{array}$$

### Uncertainty analysis

Measurement uncertainties play a significant role in evaluating the reliability of experimental results. In the present study, uncertainty analysis was conducted based on the method proposed by Hugh W. Coleman and W. Glenn Steele^35^. Table 2 shows Measurement &Calculated Parameters Uncertainty.

The uncertainty of a calculated parameter $\:R$, which is a function of several independent variables, is determined using:

19 $$\:\frac{{U}_{R}}{R}=\sqrt{{\left(\frac{\partial\:R}{\partial\:{x}_{1}}\frac{{U}_{{x}_{1}}}{R}\right)}^{2}+{\left(\frac{\partial\:R}{\partial\:{x}_{2}}\frac{{U}_{{x}_{2}}}{R}\right)}^{2}+\cdots\:}$$

**Table 2:** Parameters Uncertainty.

Parameter	Absolute Uncertainty	Measurement Relative Uncertainty	Calculated Parameters Uncertainty	Uncertainty
Temperature	± 0.1 °C	± 1%	Reynolds number	± 4–5%
Volumetric flow rate	± 1%	± 1%	Nusselt number	± 7–9%
Pressure drop	± 0.25 kPa	± 2–3%	Heat transfer rate	± 5–6%
Tube diameter	± 0.1 mm	± 1%	Pressure drop	± 6–8%
Tube length	± 1 mm	± 0.2%	Exergy efficiency	± 8–10%

The uncertainty analysis indicates that the calculated parameters fall within acceptable ranges for experimental heat transfer studies. The relatively low uncertainty values confirm the reliability of the obtained results and support the validity of the observed trends.

## Results and discussion

### Overall heat transfer performance

**Fig. 2:**
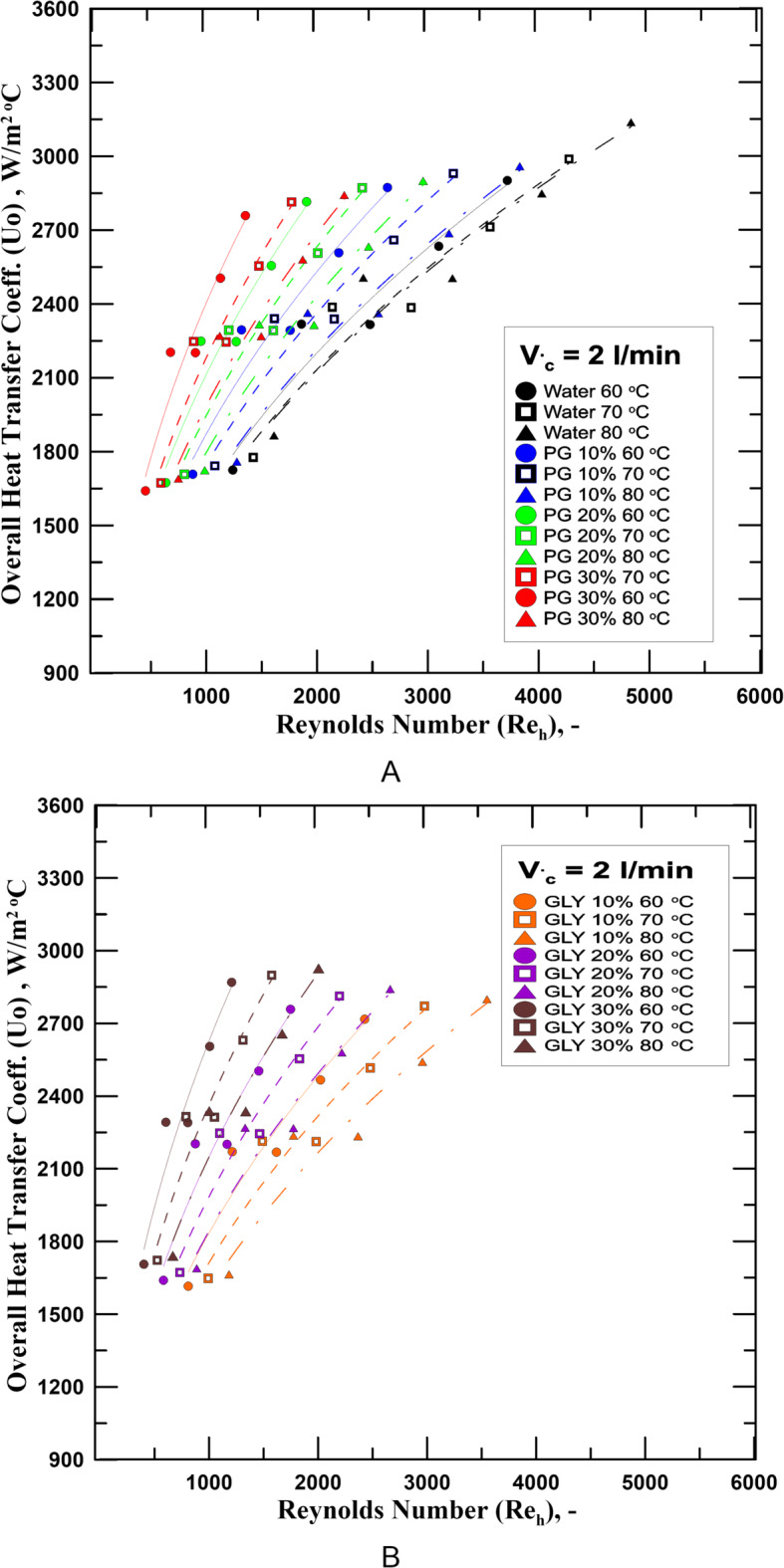
**A**. Variation of the overall heat transfer coefficient (Uo) with Reynolds number for water and propylene glycol/water mixtures at different inlet temperatures (60, 70, and 80 °C). **B**. Variation of the overall heat transfer coefficient (Uo) with Reynolds number for glycerol/water mixtures at different concentrations (10–30 wt%) and inlet temperatures (60, 70, and 80 °C).

Figure 2A and B present the variation of the overall heat transfer coefficient (Uo) with Reynolds number for all investigated fluids under counter-flow conditions. A consistent increase in Uo with Reynolds number is observed for all cases. This behavior is attributed to the enhancement of convective heat transfer as the flow velocity increases, leading to a reduction in the thermal boundary layer thickness and improved mixing within the fluid. The non-linear trend of the curves indicates that the flow regime lies within the laminar–transition region, where heat transfer enhancement becomes more sensitive to changes in flow rate.

Water exhibits the highest Uo values over the entire Reynolds number range, followed by propylene glycol (PG) mixtures, while glycerol mixtures show the lowest performance. This trend is primarily governed by fluid viscosity. Lower viscosity in water promotes higher Reynolds numbers and stronger convective effects, whereas higher viscosity in glycerol mixtures suppresses turbulence and increases thermal resistance. Within each fluid category, increasing concentration (from 10 to 30 wt%) results in a noticeable reduction in Uo. This is due to the corresponding increase in viscosity and reduction in flow effectiveness. The influence of inlet temperature is also evident, where higher temperatures lead to improved heat transfer performance due to reduced viscosity and enhanced fluid mobility.

### Hydraulic performance

**Fig. 3:**
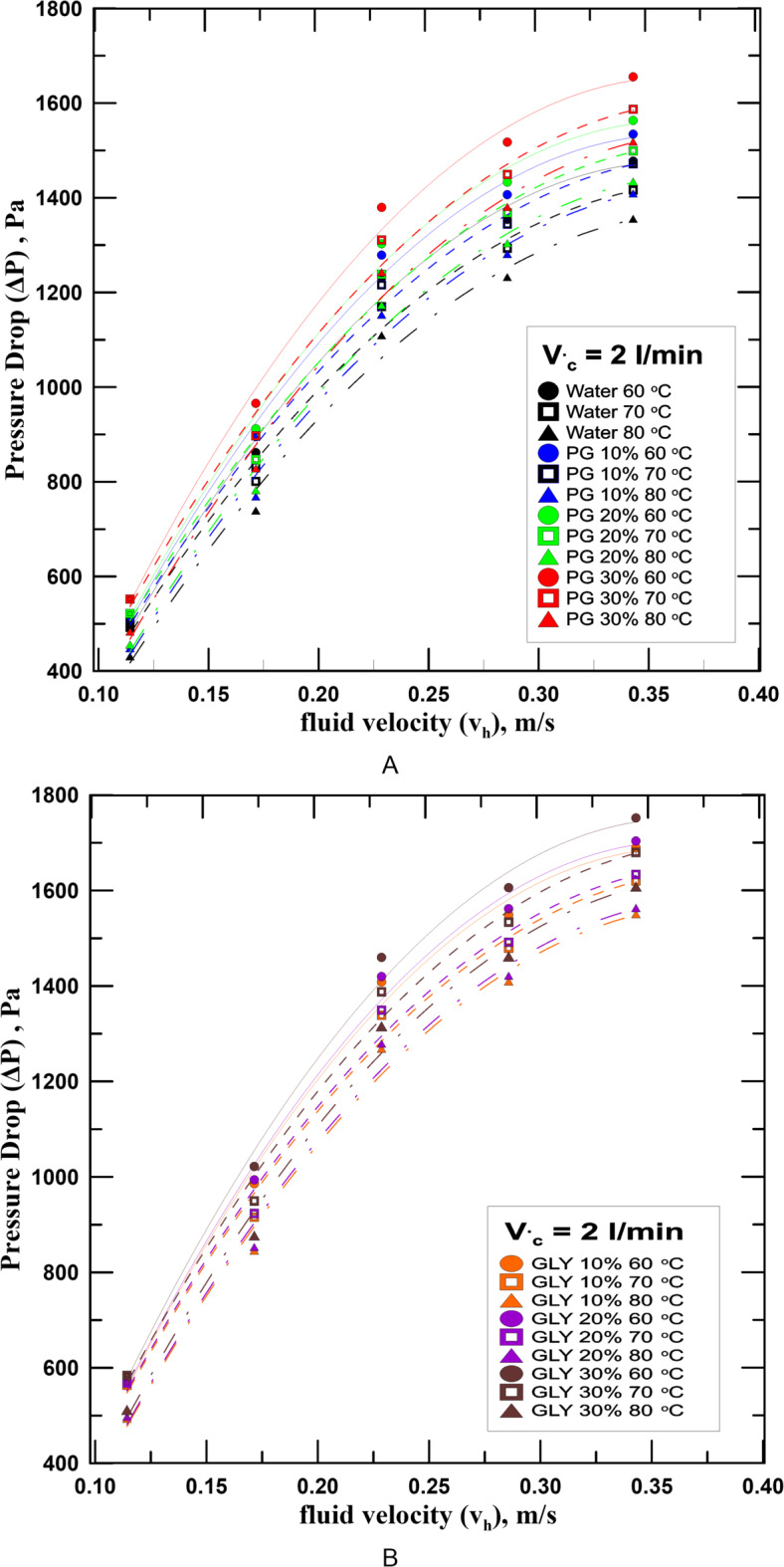
**A**. Pressure drop variation with fluid velocity for water and propylene glycol/water mixtures at different inlet temperatures under counter-flow operation. **B**. Pressure drop variation with fluid velocity for glycerol/water mixtures at different concentrations and inlet temperatures under counter-flow operation.

Figure 3A and B illustrate the variation of pressure drop (ΔP) with fluid velocity. A monotonic increase in pressure drop with increasing velocity is observed for all fluids, which is consistent with fundamental fluid dynamics behavior. As velocity increases, frictional losses inside the tubes rise significantly, resulting in higher pressure drops.

Among the tested fluids, glycerol mixtures exhibit the highest pressure drop values, followed by PG mixtures, while water shows the lowest resistance to flow. This is directly related to the increase in viscosity, which amplifies frictional forces within the flow domain. The relatively smooth and continuous nature of the curves indicates stable flow conditions and reliable experimental measurements across the investigated range.

### Convective heat transfer characteristics

**Fig. 4:**
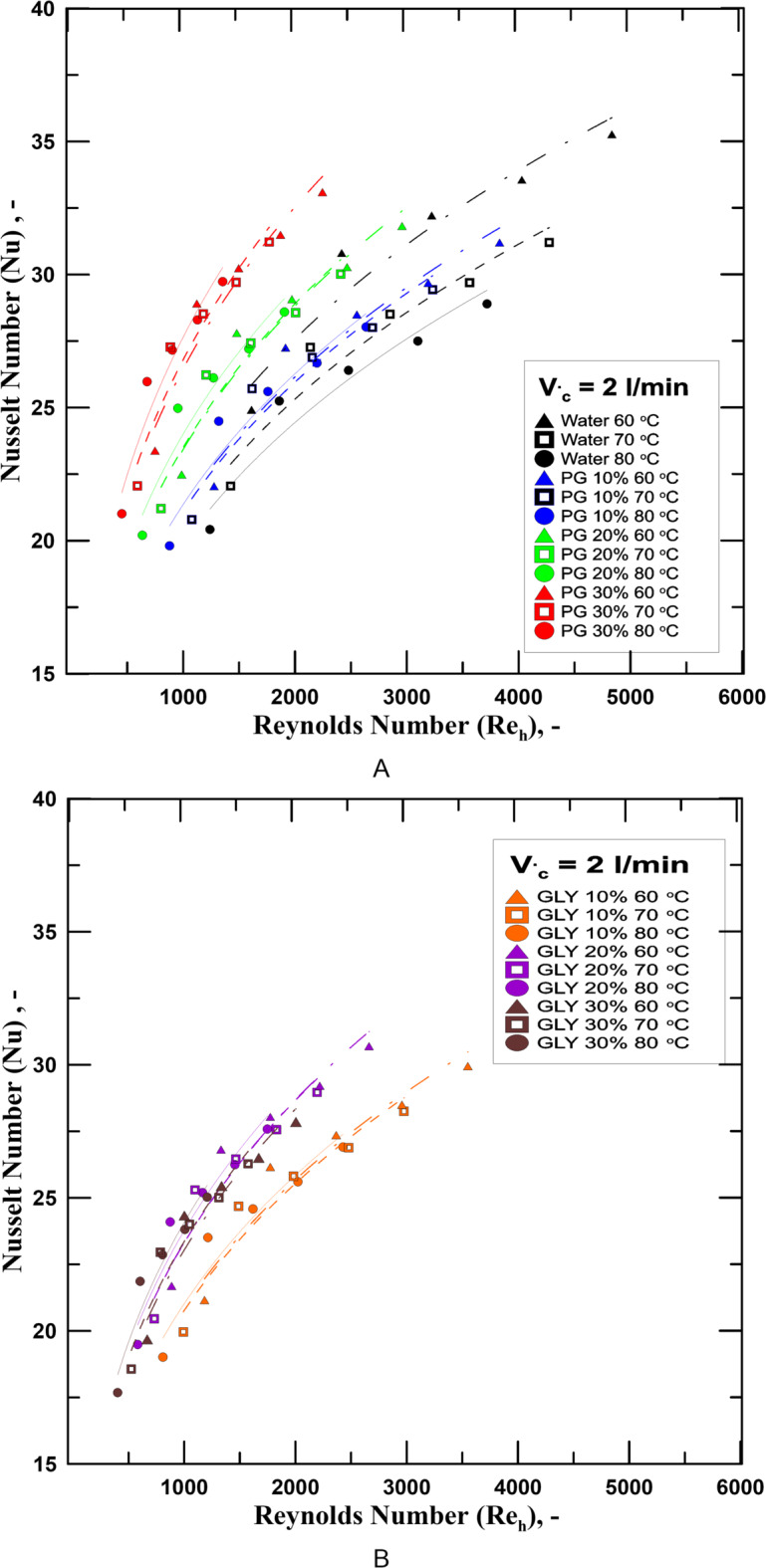
**A**. Nusselt number as a function of Reynolds number for water and propylene glycol/water mixtures at different inlet temperatures. **B**. Nusselt number as a function of Reynolds number for glycerol/water mixtures at different concentrations and inlet temperatures.

Figure 4A and B show the variation of the effective Nusselt number, calculated from the experimentally determined overall heat transfer coefficient, with Reynolds number. An increasing trend of Nu with Reynolds number is clearly observed, reflecting the enhancement of convective heat transfer with increasing flow rate. The obtained Nu values fall within the expected range for laminar to transitional flow regimes, confirming the validity of the experimental data.

Water consistently demonstrates higher Nusselt numbers compared to the tested mixtures, while glycerol solutions show the lowest values. Increasing concentration leads to a reduction in Nu, which is attributed to the adverse effect of viscosity on momentum and thermal diffusion. The results indicate that the thermal performance of the heat exchanger is strongly influenced by both flow dynamics and fluid properties.

### Friction factor analysis

**Fig. 5:**
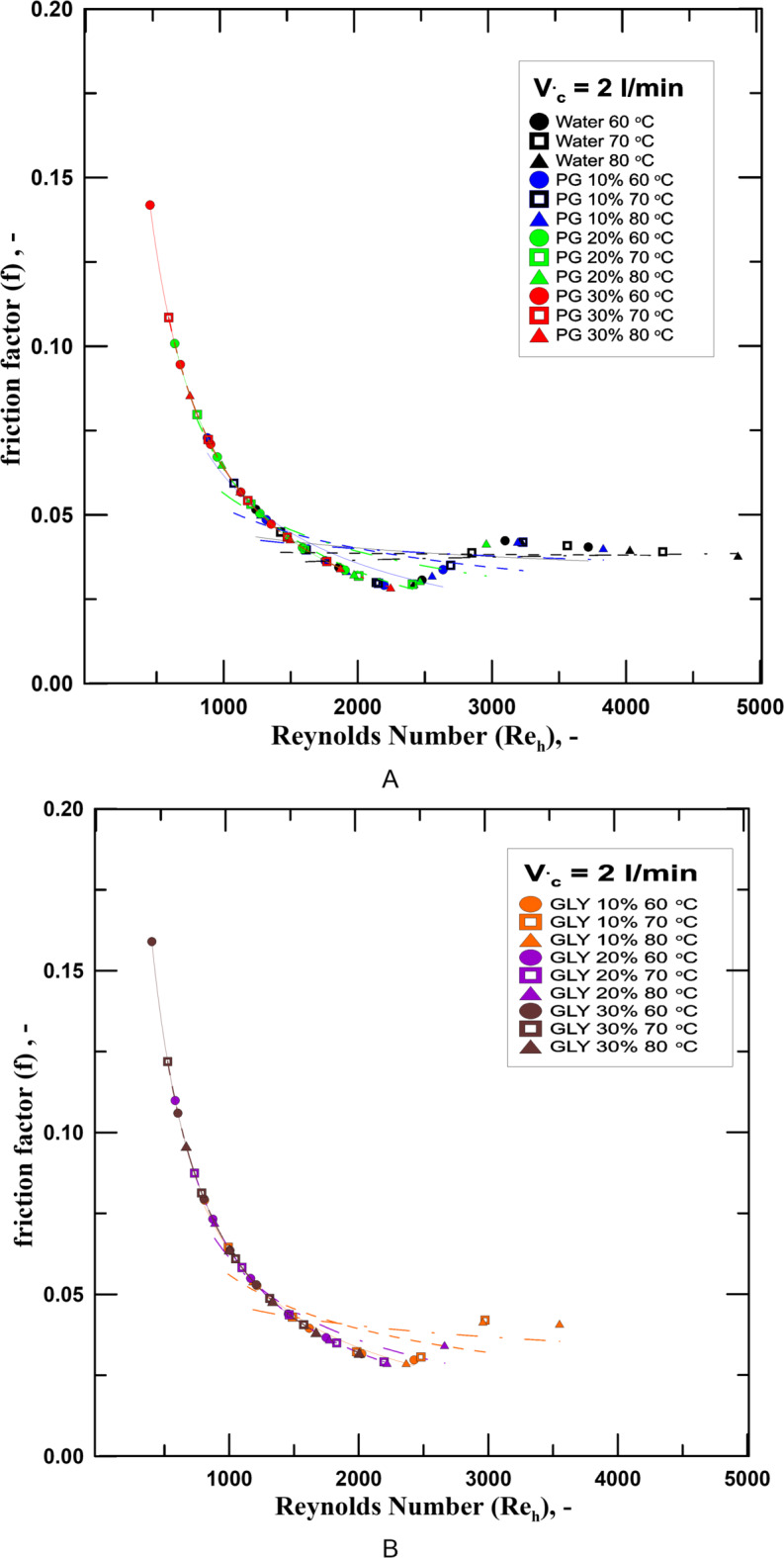
**A**. Friction factor variation with Reynolds number for water and propylene glycol/water mixtures. **B**. Friction factor variation with Reynolds number for glycerol/water mixtures.

Figure 5A and B present the variation of the Darcy friction factor (f) with Reynolds number. As expected, the friction factor decreases with increasing Reynolds number. This inverse relationship is characteristic of internal flows, particularly in the laminar and transition regimes. At lower Reynolds numbers, higher friction factors are observed due to dominant viscous effects. As the flow rate increases, inertial forces become more significant, leading to a reduction in frictional resistance. Glycerol mixtures exhibit higher friction factors compared to PG mixtures and water, which is consistent with their higher viscosity. The results show good agreement with classical fluid flow behavior in smooth tubes.

### Entropy generation analysis

**Fig. 6:**
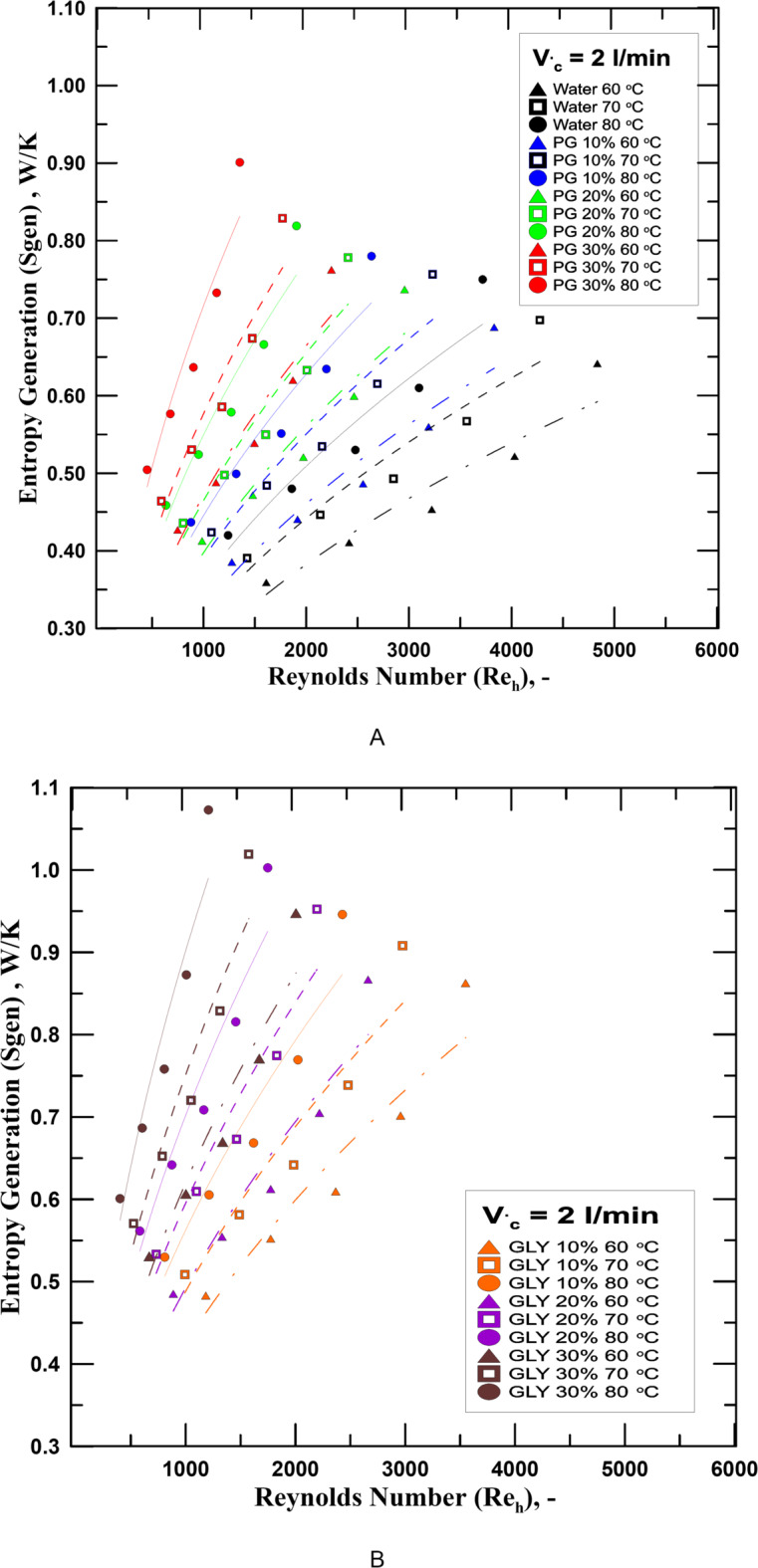
**A**. Entropy generation as a function of Reynolds number for water and propylene glycol/water mixtures. **B**. Entropy generation as a function of Reynolds number for glycerol/water mixtures.

Figure 6A and B illustrate the variation of entropy generation (S_gen_) with Reynolds number. Entropy generation increases with Reynolds number for all fluids. This trend reflects the rise in irreversibilities associated with both heat transfer and fluid friction as flow rate increases. Although higher flow rates enhance heat transfer performance, they also lead to increased viscous dissipation and thermal gradients, which contribute to higher entropy generation. This highlights the trade-off between heat transfer enhancement and thermodynamic losses. Glycerol mixtures exhibit the highest entropy generation values, followed by PG mixtures, while water shows the lowest irreversibility levels. This is attributed to the increased viscous effects in more concentrated and viscous fluids.

The obtained values fall within a physically reasonable range, confirming the consistency of the thermodynamic analysis. The obtained results indicate that Reynolds number and fluid viscosity are the most influential parameters governing entropy generation and exergy efficiency. An increase in Reynolds number enhances convective heat transfer and improves energy recovery; however, it also increases frictional irreversibilities, resulting in higher entropy generation. In addition, increasing fluid viscosity leads to larger pressure-drop penalties and internal dissipation, thereby reducing exergy efficiency. Consequently, the thermo-hydraulic behavior of the investigated fluids is primarily controlled by the combined effects of flow rate and viscosity.

### Exergy efficiency

**Fig. 7:**
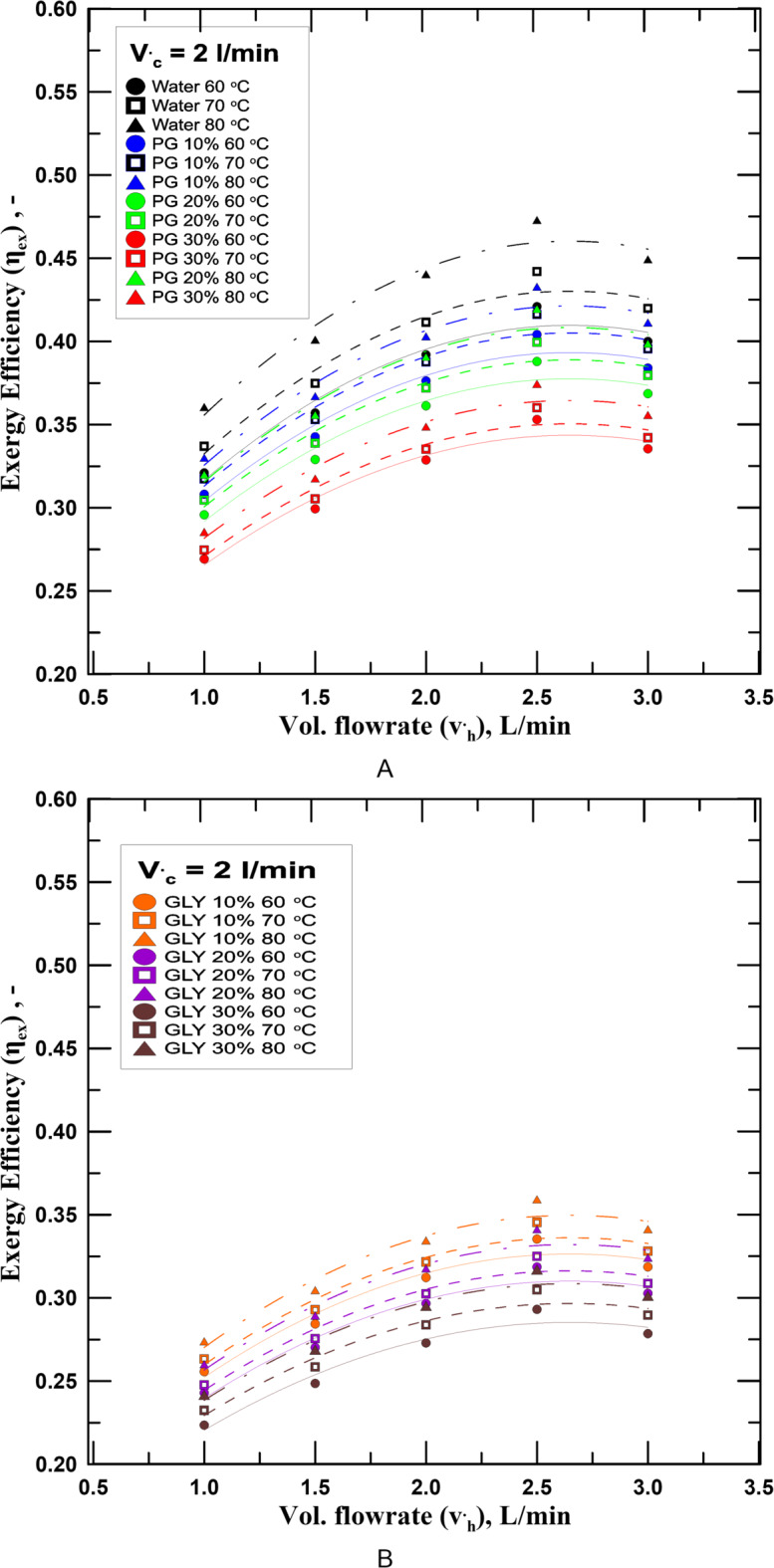
**A**. Exergy efficiency variation with hot fluid flow rate for water and propylene glycol/water mixtures. **B**. Exergy efficiency variation with hot fluid flow rate for glycerol/water mixtures.

Figure 7A and B show the variation of exergy efficiency (η_ex_) with hot fluid flow rate. An overall increase in exergy efficiency with flow rate is observed, particularly at lower flow rates. This indicates that improving heat transfer effectiveness enhances the useful energy recovery from the system. However, at higher flow rates, the rate of increase becomes less significant, suggesting the growing influence of irreversibilities. This behavior reflects the balance between improved heat transfer and increased entropy generation. Water demonstrates the highest exergy efficiency, followed by PG mixtures, while glycerol mixtures show the lowest performance. Increasing concentration leads to a reduction in exergy efficiency due to higher irreversibility losses. The obtained efficiency values fall within expected limits for compact heat exchangers operating under similar conditions.

The results demonstrate a strong coupling between fluid thermophysical properties, flow dynamics, and heat transfer performance. While increasing flow rate enhances heat transfer, it also leads to higher pressure losses and entropy generation, highlighting the importance of optimizing operating conditions. Water provides the best overall performance in terms of heat transfer efficiency and thermodynamic effectiveness, whereas the addition of viscous components such as propylene glycol and glycerol leads to a degradation in both thermal and exergy performance.

### Comparison with literature

**Fig. 8:**
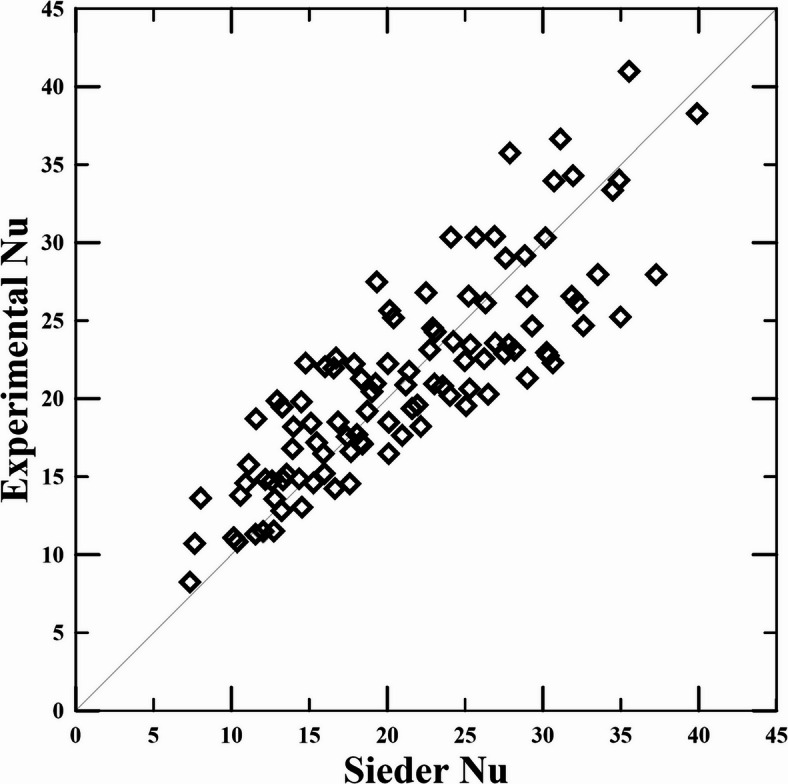
Comparison between the experimental Nusselt number and the values predicted by the Sieder–Tate correlation.

Figure 8 presents a comparison between the experimentally determined Nusselt numbers and those predicted by the classical Sieder–Tate correlation. Most of the data points are distributed close to the line of perfect agreement, indicating that the experimental measurements follow the expected heat-transfer behavior over the investigated Reynolds number range. The observed deviations may be attributed to differences in heat exchanger geometry, flow development effects, fluid-property variations, and the simplifying assumptions inherent in the correlation. Nevertheless, the overall agreement confirms the reliability of the experimental methodology and the consistency of the obtained thermal-performance data.

The present experimental results are in good agreement with previously reported studies on convective heat transfer and thermo-hydraulic performance of viscous fluid mixtures in tubular heat exchangers. The observed increase in the overall heat-transfer coefficient (Uo) and Nusselt number (Nu) with Reynolds number is consistent with the classical findings of Sieder and Tate^36^ and with the analyses of Shah and London^37^, which demonstrated the strong influence of flow conditions on heat-transfer enhancement.

The reduction in thermal performance with increasing propylene glycol and glycerol concentrations is attributed primarily to the increase in fluid viscosity, which reduces Reynolds number and weakens convective heat-transfer mechanisms. Similar behavior has been reported by Kakaç and Liu^38^, who highlighted the significant effect of viscosity on heat-transfer performance in heat-exchange systems.

Regarding hydraulic performance, the increase in pressure drop and friction factor with increasing viscosity is consistent with the Darcy–Weisbach formulation and established flow correlations for laminar and transitional regimes. The higher pressure losses observed for glycerol mixtures are therefore expected due to their higher viscous resistance.

The entropy-generation results reveal increasing irreversibilities with increasing Reynolds number as a consequence of both fluid-friction and heat-transfer effects. This trend is consistent with the second-law analyses reported by Bejan^39^, who demonstrated that enhanced heat transfer is generally accompanied by increased entropy production. Similarly, the exergy-efficiency results indicate that water provides the highest thermodynamic performance, whereas glycerol mixtures exhibit lower efficiencies due to increased viscous dissipation and irreversibility.

Overall, the agreement between the present results and established literature trends confirms the validity of the experimental procedure, data-reduction methodology, and performance analysis. Although some quantitative deviations are observed, the general behavior remains consistent with well-established heat-transfer and thermodynamic principles.

### Correlation development and validation

An empirical correlation was developed to predict the Nusselt number based on the experimental data obtained for different working fluids over the laminar–transition flow regime. A multiple regression analysis was performed using the Reynolds and Prandtl numbers, resulting in the following correlation:

$$\:Nu=2.43{\hspace{0.17em}}R{e}^{0.30}{\hspace{0.17em}}P{r}^{0.094}$$

The proposed correlation was developed using the complete experimental dataset obtained from water, propylene glycol/water mixtures, and glycerol/water mixtures. Therefore, its applicability is limited to the operating conditions investigated, namely Reynolds numbers in the range of 400 ≤ Re ≤ 4800 and the corresponding Prandtl numbers of the tested fluids. The correlation should be used only for water, propylene glycol/water mixtures (10–30 wt%), and glycerol/water mixtures (10–30 wt%) within the temperature range investigated in the present study. Extrapolation beyond these conditions may lead to increased prediction uncertainty.

**Fig. 9:**
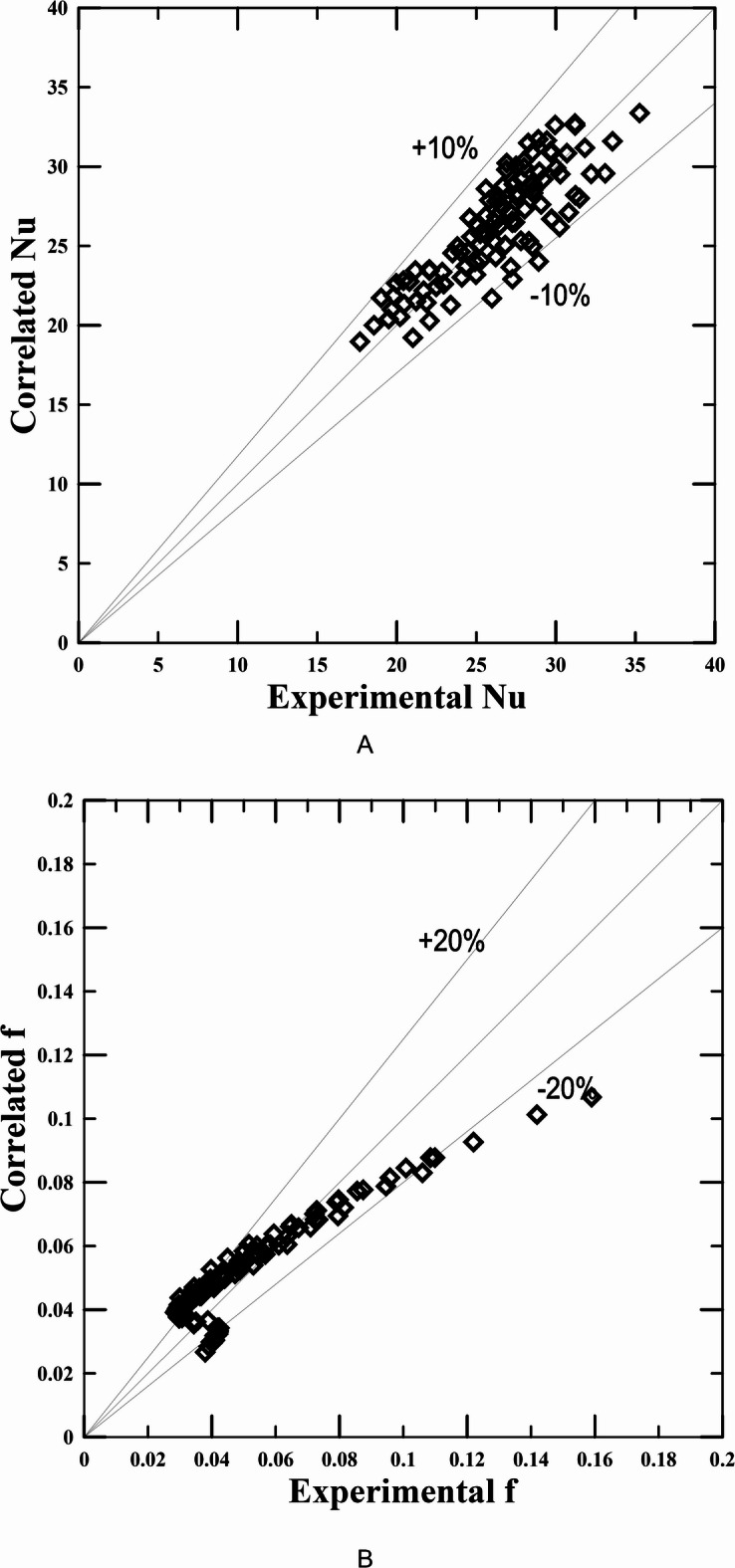
**A**. Comparison between experimental and predicted Nusselt number using the proposed correlation. **B**. Comparison between experimental and predicted friction factor using the proposed correlation.

The accuracy of the proposed correlation was evaluated by comparing the predicted Nusselt number with the experimental data, as shown in Fig. 9A. A good agreement is observed, with most data points distributed closely around the 45° line. The correlation yields a coefficient of determination of $\:{R}^{2}=0.81$and an average deviation of approximately ± 10%, confirming its reliability for predicting heat transfer performance under the present experimental conditions.

An empirical correlation was developed to predict the friction factor as a function of Reynolds and Prandtl numbers using nonlinear regression analysis of the complete experimental dataset. The proposed correlation is expressed as:

$$\:f=5.47{\hspace{0.17em}}R{e}^{-0.62}{\hspace{0.17em}}P{r}^{-0.08}$$

and is applicable within the investigated range of $\:400\le\:Re\le\:4800$for water, propylene glycol/water, and glycerol/water mixtures.

The predictive capability of the proposed correlation was evaluated by comparing the calculated friction factors with the corresponding experimental values, as shown in Fig. 9B. Most of the data points are distributed close to the line of perfect agreement and lie within the ± 20% deviation limits, demonstrating satisfactory agreement between the predicted and measured values. The relatively larger deviations observed for a limited number of data points are mainly attributed to experimental uncertainties, variations in thermophysical properties of the tested fluids, and the transition between flow regimes. Overall, the proposed correlation provides a reliable engineering tool for estimating the friction factor within the investigated operating conditions.

### Thermo-hydraulic trade-off analysis

The thermal enhancement achieved at higher Reynolds numbers is accompanied by increased hydraulic penalties. Therefore, a thermo-hydraulic assessment is necessary to evaluate the balance between heat-transfer improvement and pumping-power requirements.

The results indicate a clear trade-off between heat-transfer enhancement and hydraulic performance. Increasing Reynolds number improves the Nusselt number and overall heat-transfer coefficient, leading to enhanced thermal performance. However, this improvement is accompanied by higher friction factors and pressure drops, which increase pumping-power requirements. Therefore, the optimum operating condition should be selected based on a compromise between thermal enhancement and hydraulic penalties. This consideration becomes particularly important for viscous fluids, where the gain in heat-transfer performance may be partially offset by increased flow resistance and energy consumption.

Compared with enhanced heat-transfer techniques such as twisted tapes, corrugated tubes, plate heat exchangers, and nanofluid-based systems, the present shell-and-tube heat exchanger may provide lower heat-transfer enhancement under similar operating conditions. However, many enhancement methods are accompanied by increased pressure-drop penalties, greater manufacturing complexity, and higher operational and maintenance costs. In contrast, the shell-and-tube configuration remains one of the most reliable and widely adopted heat exchanger designs in industrial applications due to its mechanical robustness, operational flexibility, ease of maintenance, and suitability for a wide range of working fluids. Therefore, the present study focuses on understanding the influence of fluid properties on thermo-hydraulic and exergy performance within a practical industrial heat exchanger configuration. The results provide useful benchmark data for evaluating the trade-off between thermal enhancement and hydraulic penalties when selecting heat-transfer fluids for industrial applications.

## Conclusion

This study presented a comprehensive experimental investigation of the thermo-hydraulic and second-law performance of a shell-and-tube heat exchanger operating under counter-flow conditions using water, propylene glycol (PG)/water mixtures, and glycerol/water mixtures. The results demonstrated that increasing the Reynolds number significantly enhances heat transfer performance, as reflected by the increase in the overall heat transfer coefficient and Nusselt number. However, this enhancement is accompanied by a noticeable rise in pressure drop and entropy generation, indicating increased irreversibilities within the system. This confirms the inherent trade-off between thermal performance and hydraulic and thermodynamic losses.

Fluid properties were found to play a dominant role in system behavior. Water exhibited the highest heat transfer performance and exergy efficiency due to its lower viscosity, while glycerol mixtures showed the lowest performance as a result of increased viscous resistance. Increasing the concentration of viscous components led to a consistent deterioration in both thermal and exergy performance. The analysis of entropy generation revealed that irreversibility increases with flow rate, primarily due to the combined effects of fluid friction and heat transfer. Correspondingly, exergy efficiency improved with increasing flow rate at lower ranges, but showed limited gains at higher flow rates due to the growing impact of entropy generation.

An empirical correlation of the form $\:Nu=2.43R{e}^{0.3}P{r}^{0.094}\:$was developed based on the experimental data and demonstrated good agreement with the measured values, with a coefficient of determination of approximately 0.81 with most experimental data falling within an error band of ± 10%. Comparison with classical correlations confirmed that the proposed model provides more accurate predictions within the laminar–transition flow regime investigated in this study.

Overall, the findings highlight the importance of optimizing operating conditions to balance heat transfer enhancement and thermodynamic efficiency, particularly when dealing with viscous fluids. The results and proposed correlation can serve as a useful reference for the design and performance evaluation of shell-and-tube heat exchangers under similar operating conditions.

From an industrial perspective, glycol-based mixtures are widely employed as heat-transfer fluids in HVAC systems, refrigeration units, geothermal installations, solar thermal systems, and process industries due to their low freezing temperatures, elevated boiling points, and operational reliability under severe environmental conditions. However, increasing glycol concentration leads to higher fluid viscosity, which adversely affects convective heat transfer and increases pressure-drop penalties and pumping-power requirements. Therefore, although glycol-based fluids provide enhanced operational safety and freeze protection, their use requires a careful balance between thermal performance and hydraulic losses.

## Future scope and recommendations

The present study provides a comprehensive experimental assessment of the thermo-hydraulic and exergy performance of water, propylene glycol/water, and glycerol/water mixtures in a shell-and-tube heat exchanger. The results demonstrate the feasibility of employing glycol-based mixtures in applications requiring freeze protection or operation under varying environmental conditions. However, the increased viscosity of these mixtures leads to higher pressure losses and pumping-power requirements, which should be carefully considered during system design and optimization.

Future studies may investigate wider concentration ranges, higher Reynolds numbers, and different heat-exchanger configurations to further improve the understanding of fluid-property effects on thermal and hydraulic performance. Additional research is also recommended to validate the proposed empirical correlations under broader operating conditions and to evaluate the economic and thermo-economic implications of using viscous heat-transfer fluids in industrial thermal systems. Furthermore, optimization studies that simultaneously consider heat-transfer enhancement, pressure-drop penalties, and exergy performance may provide valuable guidance for the design of more efficient heat-exchange systems.

## Data Availability

The datasets used and/or analyzed during the current study are available from the corresponding author on reasonable request.

## List of symbols

A_o_: Outer heat transfer area of tubes (m²)
At: Total internal flow area of tubes (m²)
A_t,1_: Internal flow area of a single tube (m²)
C_h_: Heat capacity rate of hot fluid (W/K)
Cc: Heat capacity rate of cold fluid (W/K)
C_min_: Minimum heat capacity rate (W/K)
C_max_: Maximum heat capacity rate (W/K)
Cr: Heat capacity ratio (–)
c_p_: Specific heat capacity (J/kg·K)
d_i_: Inner tube diameter (m)
d_o_: Outer tube diameter (m)
Ė_d_: Exergy destruction rate(W)
Ė_x,h_: Exergy rate of hot fluid(W)
Ė_x,c_: Exergy rate of cold fluid (W)
f_h_: Darcy friction factor (hot fluid) (–)
g: Gravitational acceleration (m/s²)
k: Thermal conductivity (W/m·K)
L: Tube length (m)
ṁ: Mass flow rate (kg/s)
ṁ_h_: Mass flow rate of hot fluid (kg/s)
ṁ_c_: Mass flow rate of cold fluid (kg/s)
n: Number of tubes (–)
Pr_h_: Prandtl number of hot fluid (–)
Q: Average heat transfer rate (W)
Q_h_: Heat transfer rate (hot side) (W)
Q_c_: Heat transfer rate (cold side) (W)
Q_max_: Maximum possible heat transfer rate (W)
Re_h_: Reynolds number (hot fluid) (–)
Ṡ_h_: Entropy rate (hot fluid) (W/K)
Ṡ_c_: Entropy rate (cold fluid) (W/K)
Ṡ_gen_: Total entropy generation rate (W/K)
T: Temperature (K)
U_o_: Overall heat transfer coefficient (W/m²·K)
V̇: Volumetric flow rate (m³/s)
V_h_: Mean velocity of hot fluid (m/s)
Δh: Manometer height difference (m)
ΔP: Pressure drop (Pa)
ΔT_lm_: Log mean temperature difference (K)
ε: Heat exchanger effectiveness (–)
η_ex_: Exergy efficiency (–)
µ: Dynamic viscosity (Pa·s)
ρ: Density (kg/m³)
